# Learning to Dislike Chocolate: Conditioning Negative Attitudes toward Chocolate and Its Effect on Chocolate Consumption

**DOI:** 10.3389/fpsyg.2017.01468

**Published:** 2017-08-29

**Authors:** Yan Wang, Guosen Wang, Dingyuan Zhang, Lei Wang, Xianghua Cui, Jinglei Zhu, Yuan Fang

**Affiliations:** ^1^School of Psychology and Cognitive Science, East China Normal University Shanghai, China; ^2^Department of Music, East China Normal University Shanghai, China

**Keywords:** evaluative conditioning, chocolate consumption, explicit attitudes, implicit attitudes, chocolate evaluation

## Abstract

Evaluative conditioning (EC) procedures can be used to form and change attitudes toward a wide variety of objects. The current study examined the effects of a negative EC procedure on attitudes toward chocolate, and whether it influenced chocolate evaluation and consumption. Participants were randomly assigned to the experimental condition in which chocolate images were paired with negative stimuli, or the control condition in which chocolate images were randomly paired with positive stimuli (50%) and negative stimuli (50%). Explicit and implicit attitudes toward chocolate images were collected. During an ostensible taste test, chocolate evaluation and consumption were assessed. Results revealed that compared to participants in the control condition, participants in the experimental condition showed more negative explicit and implicit attitudes toward chocolate images and evaluated chocolate more negatively during the taste test. However, chocolate consumption did not differ between experimental and control conditions. These findings suggest that pairing chocolate with negative stimuli can influence attitudes toward chocolate, though behavioral effects are absent. Intervention applications of EC provide avenues for future research and practices.

## Introduction

Humans have a strong preference for high-fat foods which might readily lead to overconsumption of caloric-dense foods in the current food-rich environment (Cohen and Farley, 2008). Social psychological research concerning attitudes provides avenues for measuring and changing preferences toward unhealthy foods, and for monitoring the corresponding behavior consequences (e.g., Olson and Fazio, 2001; Hollands et al., 2011).

Attitudes, or the evaluations of various objects, are considered as one of the most important drivers of human behavior (Sweldens et al., 2014). Dual-process theories in social psychology, such as the associative-propositional evaluation (APE) model, distinguish between explicit evaluations and implicit evaluations (Gawronski and Bodenhausen, 2006, 2014). The former can be measured with traditional self-report scales, based on the assumption that explicit evaluations are consciously controlled. However, implicit evaluations are usually measured with indirect methods, for they result from automatic processes that may occur outside of people’s awareness or control. Research showed that explicit attitudes and implicit attitudes had mutual incremental validity in predicting behaviors across a wide variety of domains (Greenwald et al., 2009). As one of the most basic ways to create and change attitudes toward almost any attitude object, evaluative conditioning (EC) effect refers to the change in the valence of stimuli (conditioned stimuli, CSs) after pairing them with positive or negative valenced stimuli (unconditioned stimuli, USs) (Stahl et al., 2009). Evaluative conditioning effect has been demonstrated across a wide variety of domains including brand attitudes (Sweldens et al., 2010), self-esteem (Dijksterhuis, 2004), body satisfaction (Martijn et al., 2013), alcohol (Houben et al., 2010a), and food (Hollands et al., 2011). A meta-analysis of 214 studies revealed a medium average effect size (*d* = 0.52), confirming that EC effect is a genuine phenomenon (Hofmann et al., 2010).

Previous studies showed that EC-based interventions can change health or food-related attitudes, using positive or negative flavors as USs (Verhulst et al., 2006), using normal or obese body shapes as USs (Dwyer et al., 2007), and using aversive images as USs (Hollands et al., 2011). Due to the accumulated evidence concerning the importance of implicit processes in health behavior (for reviews, see Hofmann et al., 2008; Sheeran et al., 2013; Avishai-Yitshak and Sheeran, in press), recent studies using EC procedures to change food attitudes and behaviors mostly focused on implicit attitudes. For example, one study used a picture–picture EC procedure wherein images of snacks (e.g., chocolate, biscuits, cake, and crisps) were paired with aversive health-related images (e.g., obesity, arterial disease, and heart surgery) in the experimental condition, and images of snack foods appeared alone in the control condition (Hollands et al., 2011). Results showed that the EC procedure significantly reduced implicit attitudes toward snacks but had no effect on explicit attitudes. Furthermore, participants in the experimental condition were less likely to choose snacks as opposed to fruits, and the effect of the EC procedure on food choice behavior was partially mediated by changes in implicit attitudes. Similarly, pairing images of food with images of negative health consequences led to more healthy food choices (Hollands and Marteau, 2016). However, these findings were obtained irrespective of whether negative images were paired with snacks or fruit. These results are more consistent with a priming than an EC explanation. Another study also found that pairing pictures of snacks with negatively valenced body shapes and pairing pictures of fruits with positively valenced body shapes changed implicit attitudes toward snacks (Lebens et al., 2011). However, this study did not find behavioral change in a virtual supermarket task.

Given the inconsistent findings regarding the implicitly changed attitudes through EC procedures on subsequent food-related behaviors, it is necessary to reconsider the effectiveness of interventions targeting implicit attitudes. Furthermore, although EC effects were evident both on explicit attitudes (e.g., Jones et al., 2009) and implicit attitudes (e.g., Gawronski and LeBel, 2008), evidence of both the behavioral effect and the underlying processes is limited. Some researchers assumed that EC effects were due to automatic formation of associations in memory and thus implicit attitudes would change after an EC procedure, while explicit attitudes would not necessarily change (e.g., Gawronski and Bodenhausen, 2006, 2014). However, EC effects could also be due to controlled use of propositional knowledge about stimulus contingencies (e.g., De Houwer, 2009). In fact, a meta-analytic study revealed that EC effects were smaller with implicit measures of attitude than with other measures, such as explicit measures or choice measures, thus favoring propositional accounts (Hofmann et al., 2010).

From a practical perspective, it is important to change food choice or consumption behaviors, not just to change attitudes toward food. Existent EC studies in health psychology mainly explored food choice (which to eat) rather than food consumption (how much to eat), and led to mixed results (Hollands et al., 2011; Lebens et al., 2011; Walsh and Kiviniemi, 2014; Hollands and Marteau, 2016). To our knowledge, there were studies using modified implicit association test (IAT) to change implicit evaluations of food and subsequent consumption (e.g., Ebert et al., 2009; Haynes et al., 2015a), using attentional retraining to influence attentional bias for chocolate cues and chocolate intake (e.g., Kemps et al., 2014), yet little research has used EC procedure to change food consumption. In one study, an EC procedure pairing unhealthy food words with either positive or negative pictures was found to influence subsequent snack intake, but this effect was only observed among participants low in inhibitory control (Haynes et al., 2015b). One limitation of this study is that it contrasted snack positive with snack negative conditions and lacked a control condition. On the other hand, EC procedures have been used to manipulate attitudes and consumption behavior in other health domains, such as drinking and smoking. In one study, beer-related pictures were paired with negative words and pictures, and this procedure caused reduction of alcohol-related attitudes as well as drinking behavior for up to a week after the procedure (Houben et al., 2010b). Another study paired smoking-related stimuli with positively versus negatively valenced stimuli and found that this manipulation affected implicit attitudes toward smoking, which led to changes in explicit attitudes and self-reported smoking behavior (Măgurean et al., 2015). These studies suggested that EC procedures might affect food consumption.

In summary, although EC effects on attitudes (including food-related attitudes) were found, the effect on food consumption behavior has been less studied. The current study investigated the effects of an EC procedure on female participants’ chocolate attitudes and consumption. Chocolate was chosen because it is a popular palatable but high-calorie food, and it is also one of the most liked and craved food, especially among females (Rozin et al., 1991). Based on the studies comparing different ways of CS–US pairings in producing EC effects (e.g., Sweldens et al., 2010; Gawronski et al., 2015), we adopted simultaneous pairings of a CS with multiple USs of the same valence. In the experimental condition, pictures of chocolate were consistently paired with negative affective stimuli and pictures of fruit were consistently paired with positive affective stimuli. In the control condition, pictures of chocolate and fruit were randomly paired with both positive and negative affective stimuli. Evaluative conditioning effects on attitudes toward CSs (i.e., chocolate images versus fruit images) were assessed with self-reported ratings and IAT (Greenwald et al., 1998). We also measured participants’ awareness of the CS–US co-occurrence (i.e., contingency awareness) to examine its potential role in the EC effects. Finally, during a bogus taste test, evaluation and consumption of chocolate were measured. Thus, the outcome variables include explicit and implicit attitudes toward chocolate images, chocolate evaluation, and chocolate consumption. Given the procedural characteristic of our EC manipulation (Stahl et al., 2009; Whitfield and Jordan, 2009), we predicted that the current EC procedure would influence both explicit attitudes and implicit attitudes. We further predicted that chocolate evaluation and consumption would be influenced.

## Materials and Methods

### Participants

Participants were 102 female undergraduate students who took part for course credit or monetary compensation (approximately US$4). The mean age was 21.99 years (*SD* = 2.93 years). The mean BMI was 19.91 (*SD* = 1.88). Participants were randomly assigned to one of two conditions: experimental (*n* = 52) and control (*n* = 50).

### Procedure

Participants took part in the study individually. Informed consent was obtained after participant arrived at the laboratory. All participants were tested between 10:00 and 12:00 a.m. or between 3:00 and 5:00 p.m. to minimize initial differences in hunger. Each participant was greeted by a female experimenter and was seated at a separate room equipped with a computer. First, participants undertook an EC procedure. Next, they completed the explicit measure and the implicit measure. After that, contingency awareness during the conditioning procedure was measured with recognition memory tests. Then, after a filler task, participants tasted and rated several plates of chocolates, and the amount eaten by each participant was recorded. Afterward, participants indicated when and what they last ate before the experiment, their hungry levels before the taste test (on a 6-point Likert scale ranging from 1 = not hungry at all to 6 = extremely hungry), the importance of reducing chocolate intake (on a 6-point Likert scale ranging from 1 = not important at all to 6 = extremely important), and personal information (i.e., age, height, and weight). Finally, participants described what they thought the purpose of the experiment was. Participants were debriefed via email after data collection had been completed. This study was approved by University Committee on Human Research Protection of East China Normal University.

### EC Procedure

Five images of chocolate and five images of fruit downloaded from online sources were used as CSs. The images were digital colored photographs displayed on a white background and were similar on brightness and visual complexity. The size of the images was standardized by scaling and/or cropping them to 300 × 225 pixels. The pictures of chocolate and fruit were matched on ratings of valence (*M*_chocolate_ = 5.60, *SD* = 0.20; *M*_fruit_ = 5.71, *SD* = 0.20) and arousal (*M*_chocolate_ = 4.42, *SD* = 0.12; *M*_fruit_ = 4.50, *SD* = 0.16). These ratings were obtained from a pilot study, in which an undergraduate student sample of 30 women rated 20 chocolate images and 20 fruit images on 9-point pleasure and arousal scales. As USs, we used 25 positive and 25 negative images from the International Affective Picture System (IAPS; Lang et al., 2008). The positive USs and negative USs were matched on ratings of arousal (*M*_Postive_ = 5.73, *SD* = 0.39; *M*_negative_ = 5.78, *SD* = 0.41).

Participants were told to pay close attention to the pictures on the screen and that they would be asked a number of questions about the pictures later (Gawronski et al., 2015). Each of the 10 CSs was paired with five different USs of the same valence, and each pairing was presented twice. Therefore, the total procedure consisted of 100 CS–US parings (Stahl et al., 2009). During each trial, CS and US appeared on the screen simultaneously for 2000 ms. The inter-trial interval was 1500 ms. For participants in the experimental condition, chocolate images were paired with negative-valenced USs and fruit images were paired with positive-valenced USs. For participants in the control condition, both chocolate and fruit images were paired with positive-valenced USs (50%) and negative-valenced USs (50%).

### Measures

#### Explicit Measure

Explicit attitudes toward chocolate and fruit images were measured with two items (attractiveness and pleasantness; Pleyers et al., 2007). Participants evaluated each CS on a scale from 1 to 7. Responses to the two items were averaged to create a single explicit attitude index for each CS (Cronbach’s α = 0.932). Then, mean explicit attitudes for five chocolate images were computed and mean explicit attitudes for five fruit images were computed. In line with previous research (Hollands et al., 2011), explicit attitude scores for fruit were subtracted from those for chocolate to obtain an overall explicit attitude score, with a positive score indicating a relative preference for chocolate.

#### Implicit Measure

Implicit attitudes toward chocolate versus fruit images were measured with a chocolate versus fruit variant of the IAT (Greenwald et al., 1998). Participants’ task was to classify pictures and words that appeared in the center of the screen into the relevant categories by pressing the left (“*E*”) or right (“*I*”) keys. The appropriate category labels were presented in the bottom left side and bottom right side of the computer screen during each trial. The target categories were “chocolate” and “fruit” and the attribute categories were “good” and “bad.” The target stimuli were five pictures of the same chocolate brand (Hershey) as used in the product taste test, and five fruit pictures. They are the CSs used in the EC procedure. The attribute stimuli were five positive words and five negative words adapted from a previous study (Greenwald et al., 1998).

Participants completed a standard 7-block IAT. Blocks 1 (20 trials), 2 (20 trials), and 5 (40 trials) were practice blocks where participants practiced sorting the attributes and categories separately. Blocks 3 (20 trials), 4(40 trials), 6 (20 trials), and 7 (40 trials) were combined blocks where participants categorized stimuli according to both concepts and attributes. For the congruent combined blocks, chocolate pictures and good words were sorted on one key, while fruit pictures and bad words were sorted on another key. For the incongruent combined blocks, chocolate pictures and bad words were sorted on one key, while fruit pictures and good words were sorted on another key. The order of congruent and incongruent blocks was counterbalanced between the subjects. The IAT score for each participant was computed using the *D* measure with 600-ms error penalty (Greenwald et al., 2003), with a more positive value indicating a more positive implicit attitude toward chocolate than toward fruit images.

#### Contingency Awareness

Two types of contingency awareness were assessed for CS–US pairings during the conditioning phase with recognition memory tests (Stahl et al., 2009). Valence awareness was defined as the awareness of the valence of the USs with which a CS was paired, and identity awareness was defined as the awareness of the identity of the USs with which a CS was paired. Firstly, participants indicated for each CS the valence of USs with which it had been paired with: pleasant, unpleasant, or don’t know. Secondly, six USs of the same valence were presented, and participants indicated which one had been paired with a given CS. For each CS, awareness for US identity was probed five times, once for each of the five USs with which it had been paired. All participants completed the memory tests. However, only data in the experimental condition were analyzed, for each CS regularly co-occurred with multiple USs of the same valence in this condition.

#### Chocolate Evaluation and Consumption

During an ostensible taste and rate task, each participant was provided with 120 separately wrapped (5 g) chocolate of a well-known brand (Hershey) with different flavors. Participants were given 8 min to taste and rate them on a questionnaire containing 28 questions. Interspersed among the 28 items were two 6-point Likert items (likeability and intention to purchase, Cronbach’s α = 0.780) measuring evaluation of the chocolate (Pleyers et al., 2007; Gawronski and LeBel, 2008). After time had expired, chocolate was removed from the participant’s desk and sent to another room. The amount eaten by each participant was recorded.

#### Demand Awareness

As a check for demand awareness, participants described the purpose of the study in their own words. Five participants displayed demand awareness, that is, they were able to deduce that the conditioning procedure intended to influence their subsequent evaluation or consumption of the CSs (Jones et al., 2009). When the main analyses were repeated without the five participants, the main pattern of results did not change.

### Results

Independent sampled *t*-tests revealed no significant differences between conditions on age, hunger, BMI, and the importance of reducing chocolate intake, indicating that the randomization was successful.

#### Conditioning Effect on Explicit Attitudes and Implicit Attitudes toward Chocolate Versus Fruit Images

**Table 1** presents descriptive statistics for explicit and implicit attitudes, separately for each condition. Compared to participants in the control condition, participants in the experimental condition showed less favorable explicit [*t*(100) = 6.382, *p* < 0.001, *d* = 1.28] and implicit attitudes [*t*(100) = 2.379, *p* = 0.019, *d* = 0.48] toward chocolate versus fruit images.

**Table 1:** Effect of conditioning on explicit attitudes and implicit attitudes.

Condition	Explicit attitudes		Implicit attitudes	
	*M*	*SD*	*M*	*SD*
Control	0.60	1.00	-0.02	0.48
Experimental	-1.43	2.02	-0.24	0.46


*Positive scores indicated relative preferences for chocolate over fruit images*.

#### Conditioning Effect on Chocolate Evaluation and Consumption

**Table 2** presents descriptive statistics for chocolate evaluation and consumption during the taste test, separately for each condition. Compared to participants in the control condition, those in the experimental condition showed less favorable evaluation of chocolate, *t*(100) = 2.036, *p* = 0.044, *d* = 0.41. Nevertheless, participants in the experimental condition ate similar amount of chocolate than those in the control condition, *t*(100) = 0.802, *p* = 0.424, *d* = 0.16.

**Table 2:** Effect of conditioning on chocolate evaluation and consumption.

Condition	Chocolate evaluation		Chocolate consumption (g)	
	*M*	*SD*	*M*	*SD*
Control	4.92	0.93	37.70	29.13
Experimental	4.53	0.99	33.55	22.74


### Correlations between the Outcome Variables

**Table 3** provides Pearson’s correlations between the outcome variables. Explicit attitudes had a significant positive correlation with implicit attitudes (*r* = 0.41, *p* < 0.001) and chocolate evaluation (*r* = 0.21, *p* = 0.040), and a marginally significant positive correlation with chocolate consumption (*r* = 0.18, *p* = 0.078). In addition, implicit attitudes significantly correlated with chocolate evaluation (*r* = 0.49, *p* < 0.001) and marginally significantly correlated with chocolate consumption (*r* = 0.17, *p* = 0.084). Finally, chocolate evaluation significantly correlated with chocolate consumption (*r* = 0.23, *p* = 0.020).

**Table 3:** Correlations among the outcome variables.

Variable	1	2	3	4
(1) Explicit attitudes	—	0.41*^∗∗^*	0.21*^∗^*	0.18^†^
(2) Implicit attitudes	0.41*^∗∗^*	—	0.49*^∗∗^*	0.17^†^
(3) Chocolate evaluation	0.21*^∗^*	0.49*^∗∗^*	—	0.23*^∗^*
(4) Chocolate consumption	0.18^†^	0.17^†^	0.23*^∗^*	—


*N = 102*.

*^∗∗^p < 0.01; ^∗^p < 0.05;*^†^*p < 0.10*.

### Contingency Awareness

Valence awareness was computed as the proportion of correctly indicated US valence for participants in the experimental condition. Mean awareness of US valence was *M* = 0.91, which was significantly above chance, *t*(51) = 19.712, *p* < 0.001. Valence awareness correlated negatively with explicit attitudes, *r* = -0.373, *p* = 0.006. This indicates that participants who were better at identifying the valence of USs paired with a given CS showed less favorable explicit attitudes toward chocolate images. Valence awareness did not correlate with the other outcome variables, all *r*s < 0.15, all *p*s > 0.30.

Identity awareness was computed as the proportion of correctly selected USs for participants in the experimental condition. Mean awareness of US identity was *M* = 0.24, which was significantly above chance, *t*(51) = 5.174, *p* < 0.001. Identity awareness correlated negatively with explicit attitudes, *r* = -0.348, *p* = 0.012. This indicates that participants who were better at identifying the identity of USs paired with a given CS showed less favorable explicit attitudes toward chocolate images. Identity awareness did not correlate with the other outcome variables, all *r*s < 0.13, all *p*s > 0.39.

## Discussion

The present study examined whether attitudes toward chocolate and consumption of chocolate can be influenced through an EC procedure wherein chocolate images were paired with negative stimuli. Results revealed that participants in the experimental condition showed more negative explicit and implicit attitudes toward chocolate images, compared to participants in the control condition. In addition, those in the experimental condition evaluated chocolate more negatively than those in the control condition. However, the EC procedure did not influence the amount of chocolate that participants consumed. These findings have practical implications for health interventions and theoretical implications for EC research.

Given the substantial evidence concerning the predictive validity (i.e., prediction of behaviors) of implicit and explicit attitude measures (Greenwald et al., 2009), interventions targeting attitudes may have the potential to change health-related behavior. Evaluative conditioning procedures have been proved to be capable of changing attitudes (Hofmann et al., 2010), but evidence of their effects on behaviors was rather limited. Although there were studies showing that EC procedure could change drinking behavior (Houben et al., 2010a,b), the effect of EC procedure on food consumption has been less studied. Our study examined whether an EC procedure can influence attitudes toward chocolate and chocolate intake. Results showed that pairing chocolate with negative stimuli led to more negative explicit and implicit attitudes toward chocolate relative to the control condition. Moreover, the EC procedure influenced participants’ evaluation of the chocolate when tasting it. These results replicate earlier findings showing EC effects on food-related attitudes (e.g., Dwyer et al., 2007; Hollands et al., 2011) and on both explicit and implicit attitudes (for a review, see Hofmann et al., 2010). The findings suggest that EC has the potential to change attitudes toward familiar food objects (i.e., chocolate).

Nevertheless, the effect of the EC procedure on chocolate consumption was not significant. In other words, although participants conditioned to dislike chocolate showed more negative explicit and implicit attitudes toward chocolate images and evaluated chocolate more negatively than participants in the control condition, they did not consume less of chocolate. Moreover, there were marginally significant though far from perfect correlations between attitudes and consumption. These results suggest that attitudes are not the sole or necessarily the most important determinants of behavior. One possible explanation for failing to obtain behavioral effect can be found in the theory of reasoned behavior (Ajzen, 1991). According to this theory, attitudes, norms, and self-efficacy predict an individual’s intention to engage in a specific behavior, which is the most proximal determinant of actual behavior. Experimental evidence supports the idea that changing these elements promotes health behavior change (Sheeran et al., 2016). In the present study, participants’ chocolate consumption might be more influenced by inferred or implied rules about how much they should consume during a taste test (i.e., norms) than by their personal attitudes toward chocolate. Further research is needed to disentangle the effects of attitudes and norms on food consumption. A second explanation could be that changed attitudes may not necessarily translate into changed behavior for all individuals across all situations. Indeed, one study has found that an EC procedure influences unhealthy food consumption, but only among participants low in inhibitory control (Haynes et al., 2015b). Another study showed that candy consumption was predicted by automatic candy attitudes in participants depleted of self-control resources (Hofmann et al., 2007). Thus, an intervention designed to change behavior may be most likely to be effective for certain individuals (e.g., low inhibitory control) or under certain situational boundary conditions (e.g., self-control depletion). Third, the effect size of our EC procedure is smaller than what a previous meta-analysis indicated (Hofmann et al., 2010), especially when using chocolate evaluation or consumption as the outcome variable. Therefore, future studies need to investigate how best to implement the EC procedure and how to maximize behavior change effects.

There exist enduring controversies related to several functional properties of EC effect and the underlying mechanisms of EC effect (Sweldens et al., 2014). Firstly, all EC studies must exclude the possibility of demand compliance, which may lead to artifactual liking ratings that do not reflect true attitude change (De Houwer, 2011). The level of demand awareness was relatively low in the current study, and excluding demand aware participants did not alter the patterns of results. In addition, we used implicit measure of attitudes to reduce the impact of demand compliance (De Houwer, 2011). Another related but different issue is the role of contingency awareness in producing the EC effect. Some researchers suggested that EC procedure can change attitudes without participants’ awareness of the CSs–USs pairings (e.g., Jones et al., 2009), whereas others contended that EC effect only occurs when participants are aware of the CSs–USs contingencies (e.g., Stahl et al., 2009). As for the processes that underlie EC effects, some accounts focus on the automatic formation of associations in memory (e.g., Gawronski and Bodenhausen, 2006, 2014), whereas others emphasize the non-automatic use of propositional knowledge about stimulus contingencies (e.g., Mitchell et al., 2009). The current study used recognition tests of contingency awareness, which are more sensitive than open-ended questions or free recall tasks (Sweldens et al., 2014). Our study showed that EC effect on explicit attitudes was present yet attenuated when contingency awareness was low compared with when contingency awareness was high. On the other hand, the correlations between contingency awareness and EC effects on implicit attitudes, chocolate evaluation, and consumption were non-significant. These results suggest that both automatic and propositional processes were taking effects in our EC procedure, thus supporting the dual process model (De Houwer, 2009). However, there exists large diversity in EC procedures, such as simultaneous/sequential CS–US pairings, single-US/multiple-US pairings, obvious/hidden CS–US pairings (Jones et al., 2009; Hofmann et al., 2010; De Houwer, 2011). We attempted to influence attitudes toward food and the corresponding eating behavior via EC, and the processes underlying EC effects were not of primary interest. Evaluative conditioning procedures other than the current one may show different characteristics and be due to different mechanisms.

Strengths of the present study include the use of objective measure of food consumption under the taste test cover study to test the EC effect and the use of established implicit measure to reduce the demand compliance effect. Limitations of the current study should be considered. First, we used a between-subject design and operationally defined EC effect as the difference between experimental condition and control condition (Hofmann et al., 2010). Implicit attitudes, explicit attitudes, chocolate evaluation, and consumption were measured after the EC procedure. Although changes in attitudes toward chocolate from pretest to posttest could not be calculated, this design has the strength of avoiding artifactual EC effects due to repeated ratings (De Houwer, 2011). Nevertheless, future research needs to collect baseline measures of chocolate preference and examine whether pre-existing differences in preference moderate the EC effects. Second, the effects of EC procedure on attitudes and consumption were assessed immediately after the intervention. Future studies need to address the durability of the EC effects over a long period of time. Third, the current study employed only a sample of female undergraduate students, a single snack food (i.e., chocolate), and was carried out in a controlled laboratory setting. It remains to be tested whether these findings apply to other populations (children, restrained eater, clinical populations, etc.), various healthy or unhealthy foods, and different circumstances (e.g., in field settings). Finally, given that the effect of EC on chocolate consumption was not significant, integrating EC with other intervention techniques (self-control training, implementation intentions, etc.) may be more effective in changing eating behavior.

In sum, our study shows that EC procedure has the potential to influence attitudes toward familiar foods, and suggests that EC may be added to existing intervention techniques. Further research is needed to address the effectiveness, generalizability, and longevity of the training effects. Moreover, integration of EC technique into existing behavior change interventions might represent a promising future avenue of research and practices.

## Ethics Statement

This study was carried out in accordance with the recommendations of guidelines developed by the University Committee on Human Research Protection of East China Normal University with written informed consent from all subjects. All subjects gave written informed consent in accordance with the Declaration of Helsinki. The protocol was approved by the University Committee on Human Research Protection of East China Normal University.

## Author Contributions

YW and LW conceived and designed the study; YW, GW, and DZ drafted the paper; YW, GW, DZ, LW, and XC performed the experiments; YW, JZ, and YF analyzed the data.

## Conflict of Interest Statement

The authors declare that the research was conducted in the absence of any commercial or financial relationships that could be construed as a potential conflict of interest.
